# Identification and validation of *BCL6* and *VEGFA* as biomarkers and ageing patterns correlating with immune infiltrates in OA progression

**DOI:** 10.1038/s41598-023-28000-9

**Published:** 2023-02-13

**Authors:** Ziyi Chen, Wenjuan Wang, Yinghui Hua

**Affiliations:** Department of Sports Medicine, Huashan Hospital, Fudan University, No. 12, Wulumuqi Zhong Road, Shanghai, 200040 People’s Republic of China

**Keywords:** Biological techniques, Biotechnology, Genetics, Immunology, Biomarkers, Diseases

## Abstract

Osteoarthritis (OA), the most common type of arthritis, is a complex biological response caused by cartilage wear and synovial inflammation that links biomechanics and inflammation. The progression of OA correlates with a rise in the number of senescent cells in multiple joint tissues. However, the mechanisms by which senescent cells and their involvement with immune infiltration promote OA progression are not fully understood. The gene expression profiles and clinical information of OA and healthy control synovial tissue samples were retrieved from the Gene Expression Omnibus database, and then differential analysis of senescence regulators between OA and normal samples was performed. The random forest (RF) was used to screen candidate senescence regulators to predict the occurrence of OA. The reverse transcription quantitative real-time PCR experiments at tissue’s level was performed to confirm these biomarkers. Moreover, two distinct senescence patterns were identified and systematic correlation between these senescence patterns and immune cell infiltration was analyzed. The senescence score and senescence gene clusters were constructed to quantify senescence patterns together with immune infiltration of individual OA patient. 73 senescence differentially expressed genes were identified between OA patients and normal controls. The RF method was utilized to build an OA risk model based on two senescence related genes: *BCL6* and *VEGFA*. Next, two distinct aging patterns were determined in OA synovial samples. Most patients from senescence cluster A were further classified into gene cluster B and high senescence score group correlated with a non-inflamed phenotype, whereas senescence cluster B were classified into gene cluster A and low senescence score group correlated with an inflamed phenotype. Our study revealed that senescence played an important role in in OA synovial inflammation. Evaluating the senescence patterns of individuals with OA will contribute to enhancing our cognition of immune infiltration characterization, providing novel diagnostic and prognostic biomarkers, and guiding more effective immunotherapy strategies.

## Introduction

Osteoarthritis (OA) is a common degenerative disease leading to pain, joint destruction and disability with senior citizens most involved^1,2^. With an ageing population, it exerts a tremendous pressure on health-care systems and socioeconomic cost^2,3^. Joint replacement surgery is effective when OA advancing into end stage, although with a mass of limitations such as the difficulty of postoperative recovery and lifespan of prostheses^1,4,5^. Therefore, it is better to make early intervention, however, traditional early proactive management of OA consisting of pain medication could not prevent OA progresses into end stage^1^. Additionally, due to the heterogeneity of OA clinical manifestations, validated early-stage diagnostic criteria are also unavailable^1^. Therefore, an effective model to predict the risk of OA is demanded.

Research into the pathophysiology of OA has focused on cartilage and peri-articular bone. However, there is increasing recognition that OA affects all joint tissues, particularly the synovium^6^. Synovial inflammation is present in the OA joint and has been associated with radiographic changes and pain progression^7,8^. Several OA risk factors, including aging, obesity, trauma, and mechanical loading play a role in OA pathogenesis, likely by modifying synovial biology^7^. In addition, other factors, such as mitochondrial dysfunction, damage-associated molecular patterns, cytokines, metabolites, and crystals in the synovium activate synovial cells and mediate joint inflammation^7^. Therefore, a deeper understanding of the synovial molecular mechanisms associated with the occurrence and progression of OA is of great significance for developing early diagnostics and treatments for OA^9^.

It is well accepted that aging is an important contributing factor in the development of OA^10^. A rise in the number of senescent cells in joint tissues and the release of senescence-associated secretory phenotype (SASP) in cartilage degradation has been implicated in OA^11^. In joint tissue cells, age-related mitochondrial dysfunction and associated oxidative stress might induce senescence^11^. It may include an age-related pro-inflammatory state that has been termed as “inflamm-ageing”^10^.However, the mechanisms by which senescent cells contribute to OA progression are not fully understood and it remains uncertain which joint cells and SASP-factors contribute to the OA phenotype^11^.

Molecular classification of OA which is popular nowadays allows for the prediction of high-risk OA individual, the diagnosis of early OA, and the assessment of individual-based therapy^12,13^. Single cell RNA sequencing of OA synovial tissues also revealed distinct cell subtypes with different dominant function^14,15^. Nonetheless, study analyzing classification of distinct senescence clusters and analyzing its connection with immune infiltration was missing up to now. A better understanding of which senescence pattern individuals are and their association with inflammation may be critical in individual-based diagnosis, treatment, prognosis.

Here, in our study, we used gene expression profiling data of 182 senescence related genes for 74 tissues in the Gene Expression Omnibus (GEO) database to obtain the original data information. Two candidate senescence regulators (*BCL6* and *VEGFA*) were selected to predict the risk of OA using RF model. We revealed two distinct senescence expression patterns and their correlation with immune infiltration in OA synovial tissues, and constructed a senescence scoring system in clinical cohorts with large-scaled expression. Our scoring system may work as an excellent tool for treatment response and prognosis in OA patients.

## Materials and methods

### Data access and preprocessing

Gene expression matrix and its clinical information were downloaded from the GEO (http://www.ncbi.nlm.nih.gov/geo/) database. A total of 74 synovial samples were included from the GSE1919, GSE41038, GSE55235, GSE82107 and GSE55457 datasets, with 38 cases of OA patients and 36 healthy controls. To adjust the microarray, we employed the SVA method to merge the GSE1919, GSE41038, GSE55235, GSE82107 and GSE55457 datasets^16^. Also, 279 cell senescence regulator genes were obtained from the CellAge database (https://genomics.senescence.info/cells/)^17^. Finally, 182 senescence regulators were extracted to identify different senescence patterns in our study.

### Identification of differentially expressed genes (DEGs) between OA and normal

Senescence related DEGs between OA and normal were screened out using the “limma” package with the criterion setting as *P* < 0.05 and |log fold change (FC)|> 1^18,19^.

### The feature genes were screened based on the RF analysis

To predict the occurrence of OA, we constructed a training model adopting both support vector machine (SVM) and RF methods. Boxplots of residuals, reverse cumulative distribution of residuals and receiver operating characteristic (ROC) curve was used to compare the accuracy of the two models. The RF method was then selected to screen differentially express RNA modification regulators using the R library ‘randomForest’ with ‘mtry’ and ‘ntree’ setting to 3 and 500, respectively^20^. The optimal ‘ntree’ was chosen according to minimum cross-validation error in tenfold cross-validation and the significance of differentially-expressed RNA modification regulators with the optimal ntree were assessed. We then constructed a nomogram using the ‘rms’ package^21,22^. Calibration curves were used to evaluate the consistency between the observed and predicted values. Finally, we performed clinical impact curve and decision curve analyses to evaluate the clinical benefits of our model. A binary logistic regression test was conducted to evaluate the model using SPSS (version 23).

### Identification of senescence clusters

Senescence patterns were identified based on senescence DEGs using the “ConsensusClusterPlus” R package was used^23^. The principal component analysis (PCA) was conducted to correlate the principal component with senescence clusters^24^.

### Single-sample gene-set enrichment analysis (ssGSEA)

The relative infiltration levels of 23 immune cells in the GSE1919, GSE41038, GSE55235, GSE82107 and GSE55457 dataset were quantified using the ssGSEA algorithm^25^. Spearman correlations were calculated for 23 immune infiltrating cells with 73 senescence regulators, followed by visualization using the ‘ggplot2’ package^26^.

### Functional enrichment analysis

DEGs between senescence clusters were screened using the “limma” package^19^. Meanwhile, GO enrichment analyses (www.geneontology.org/) and KEGG pathway (http://www.kegg.jp/kegg/kegg1.html) were performed using the R package, including “clusterProfiler”, “org.Hs.eg.db”, “enrichplot”, “ggplot2”, “RColorBrewer”, “dplyr”, and “ComplexHeatmap”^26–34^. The *P* value < 0.05 was considered significantly enriched.

### Identification of senescence gene clusters

We first extracted the DEGs from the two senescence clusters and performed consensus clustering to generate gene clusters using the “ConsensusClusterPlus” R package^23^.

### Senescence score construction

We then constructed a scoring system to describe the senescence regulator expression pattern for individuals based on DEGs between OA and normal using PCA^24^. Principal component 1 was used for score calculation.

$${\text{m6A}}\;{\text{score}} = \sum {\text{PC1i}}$$

where i is the expression of senescence phenotype-related genes.

### Correlation between senescence gene signature and inflammation

The association between senescence clusters, gene clusters, senescence scores and immune filtration were analyzed. The abundance of 23 immune cells in the two senescence gene clusters was assessed using ssGSEA method. Gene expression of Mitogen-activated protein kinases (MAPK) pathway were compared in the two senescence and gene clusters.

### Sample collection

Synovial tissue from 3 patient of meniscus injury and 3 of OA were collected from Huashan hospital. All patients critically read and signed the informed consent form (KY2020-060) which as approved by the ethics committee of Huashan Hospital. The research followed the guidelines of the 1975 Declaration of Helsinki.

### Reverse transcription-quantitative polymerase chain reaction (RT-qPCR)

The total synovial tissue RNA was extracted using Trizol (Servicebio) and then total RNA was reverse transcribed to complementary DNA (cDNA) using Servicebio®RT Enzyme Mix. The qRT-PCR was performed using the 2 × SYBR Green qPCR Master Mix (None ROX) (Servicebio). The primer sequence of genes used in our study was listed in Table S1. Genes were normalized to GAPDH. Relative levels of mRNA were expressed as fold-changes as calculated by the 2^–ΔΔCT^ method. Each biological sample was technically performed in triplicate.

### Statistical analysis

All statistical analyses in our study were performed with R software, version 4.1.1. The Wilcoxon test was performed for groups comparisons, and *P* < 0.05 was defined as a significant difference. No multivariable analysis was conducted in our study to adjust the *P* value of each of the DEGs.

## Results

### Analysis of expression characteristics of senescence regulators in OA synovial tissues

**Figure 1:**
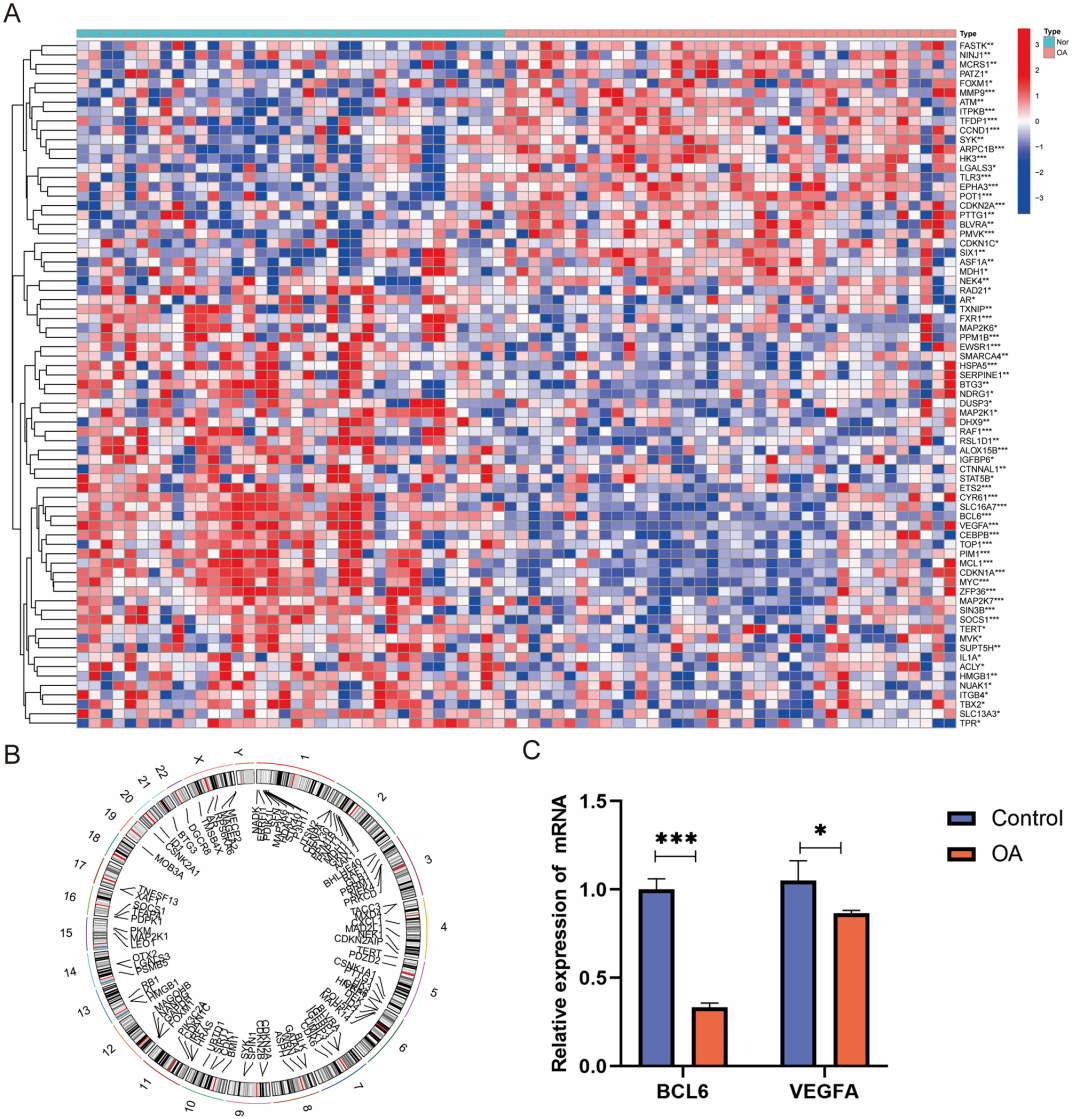
Identification of differentially expressed senescence genes in OA. (**A**) Heatmap showing the significantly different expression of senescence regulator genes in OA and normal synovial tissues. (**B**) The location of these genes on the chromosome. (**C**) The relative expression of mRNA in the normal and OA group. (All figures *represents *p* < 0.05, **represents *p* < 0.01, ***represents *p* < 0.001) OA, osteoarthritis.

A total of 73 senescence regulators were finally identified in this study. The differences in senescence gene expression between groups were visualized in heatmap (Fig. 1A). The location of senescence related genes in chromosome was shown in Fig. 1B.

### Construction of the OA predictive model using the SVM and RF methods

**Figure 2:**
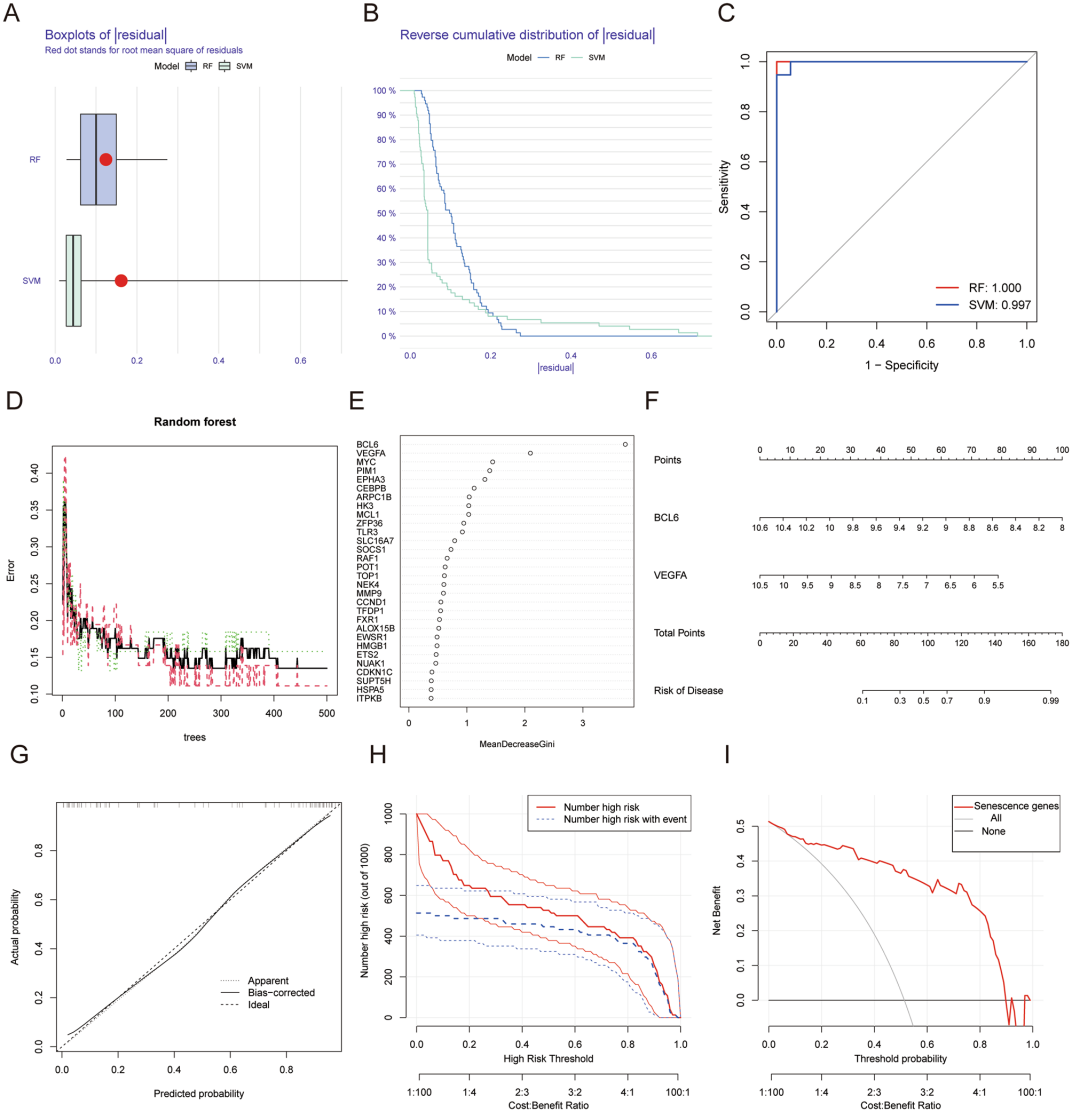
Construction of OA risk predictive model using the SVM and RF methods. Boxplot of the residual distribution (**A**) and reverse cumulative distribution of residual (**B**) as a function of the values and ROC curves (**C**) showing the observed sensitivity between RF and SVM. (**D**) RF: prediction error curves based on tenfold cross-validation. (**E**) The importance of the 30 senescence regulators based on the RF model. (**F**) Nomogram graph of the predictive model based on two m6A regulators. The calibration curves (**G**), clinical impact plot (**H**) and DCA (**I**) were used to determine the clinical utility of risk prediction nomograms. SVM, support vector machine; RF, random forest; OA, osteoarthritis; ROC, receiver operating characteristic; DCA, decision curve analysis.

The prediction performance between the SVM and RF methods were compared. “Boxplots of residual” (Fig. 2A), “reverse cumulative distribution of residual” (Fig. 2B), and a ROC curve (Fig. 2C) vealed that RF exhibited significantly high predictive capability. According to the minimum cross-validation error in tenfold cross-validation, the best ‘ntree’ was selected (Fig. 2D). In total, we identified 30 senescence regulators and ranked them according to their importance (Fig. 2E). The calibration curves, clinical impact plots and decision curve analysis (DCA) showed that the nomogram model may be an ideal predictive model for OA (Fig. 2G,H,I). The nomogram evaluation model was constructed based on two senescence regulators (*BCL6* and *VEGFA*) to predict the probability of OA (Fig. 2F). PCR results found that the mRNA levels of BCL6 (*P* < 0.001) and VEGFA (*P* < 0.05) were higher in normal tissue compared to OA which was consistent with the bioinformatics tools (Fig. 1C). The binary logistic regression analysis demonstrated that the model was a good fit, and the significance of Hosmer-lemeshaw test was 0.553 (> 0.05).

### Identification of two distinct senescence clusters

**Figure 3:**
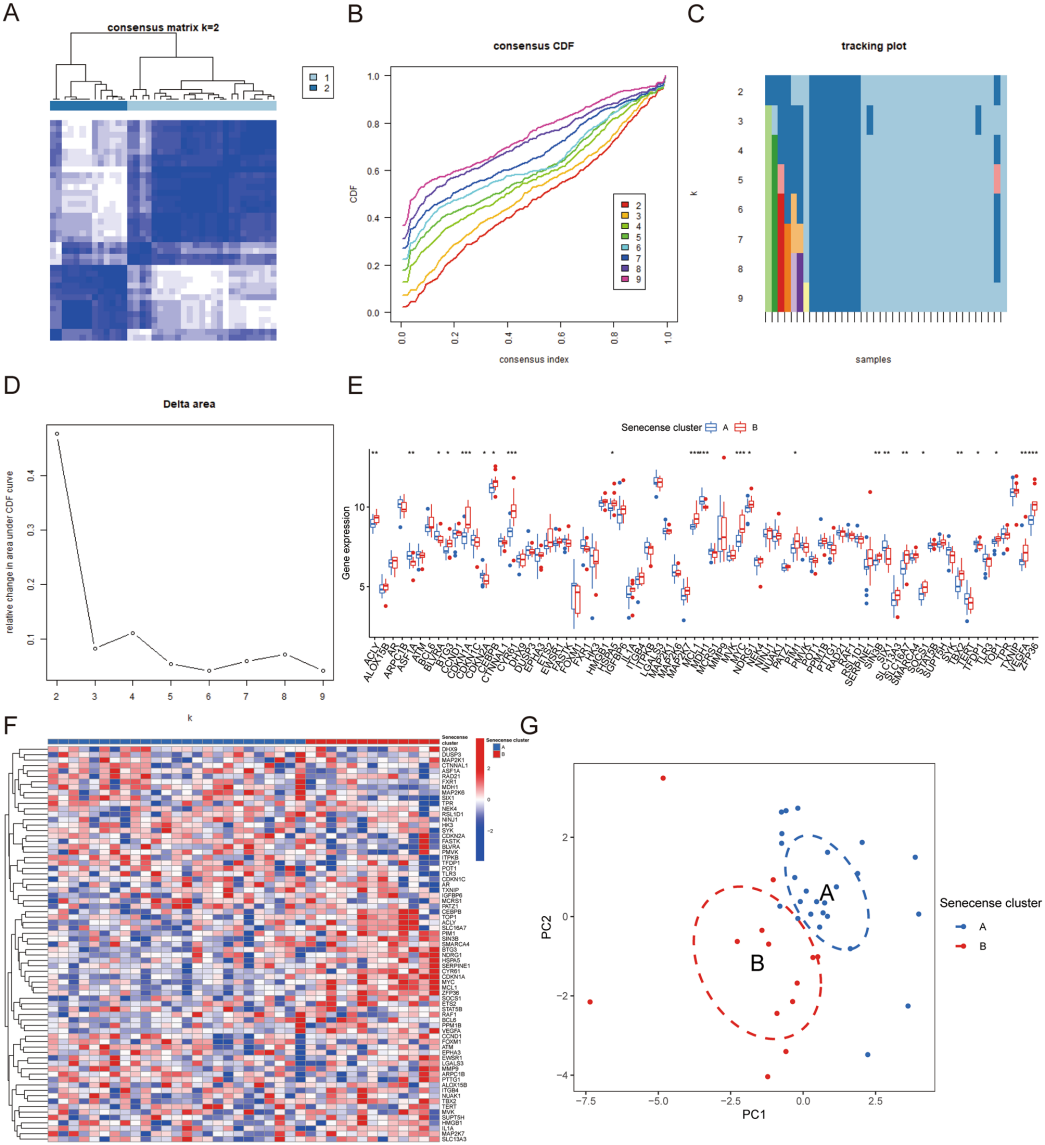
Identification of two senescence clusters. (**A**–**D**) Clustering of OA synovial samples based on senescence regulators. (**E**) Bloxplot showing the gene expression of senescence regulators. (**F**) Heatmap demonstrating the DEGs of the two senescence clusters. (**G**) PCA was used to verify the two senescence clusters. (All figures *represents *p* < 0.05, **represents *p* < 0.01, ***represents *p* < 0.001) OA, osteoarthritis; DEGs, differentially expressed genes; PCA, principal component analysis.

Two senescence clusters were identified (senescence cluster A and B) based on 73 senescence DEGs between OA and normal synovial samples using consensus clustering (Fig. 3A–D). The boxplot and heatmap demonstrated the 73 senescence genes’ expression in the two distinct ageing groups (Fig. 3E, F). PCA was applied to verify the two distinct ageing clusters divided by consensus clustering of the 73 senescence regulators (Fig. 3G).

### Immune cell infiltration analysis in the two senescence phenotypes

**Figure 4:**
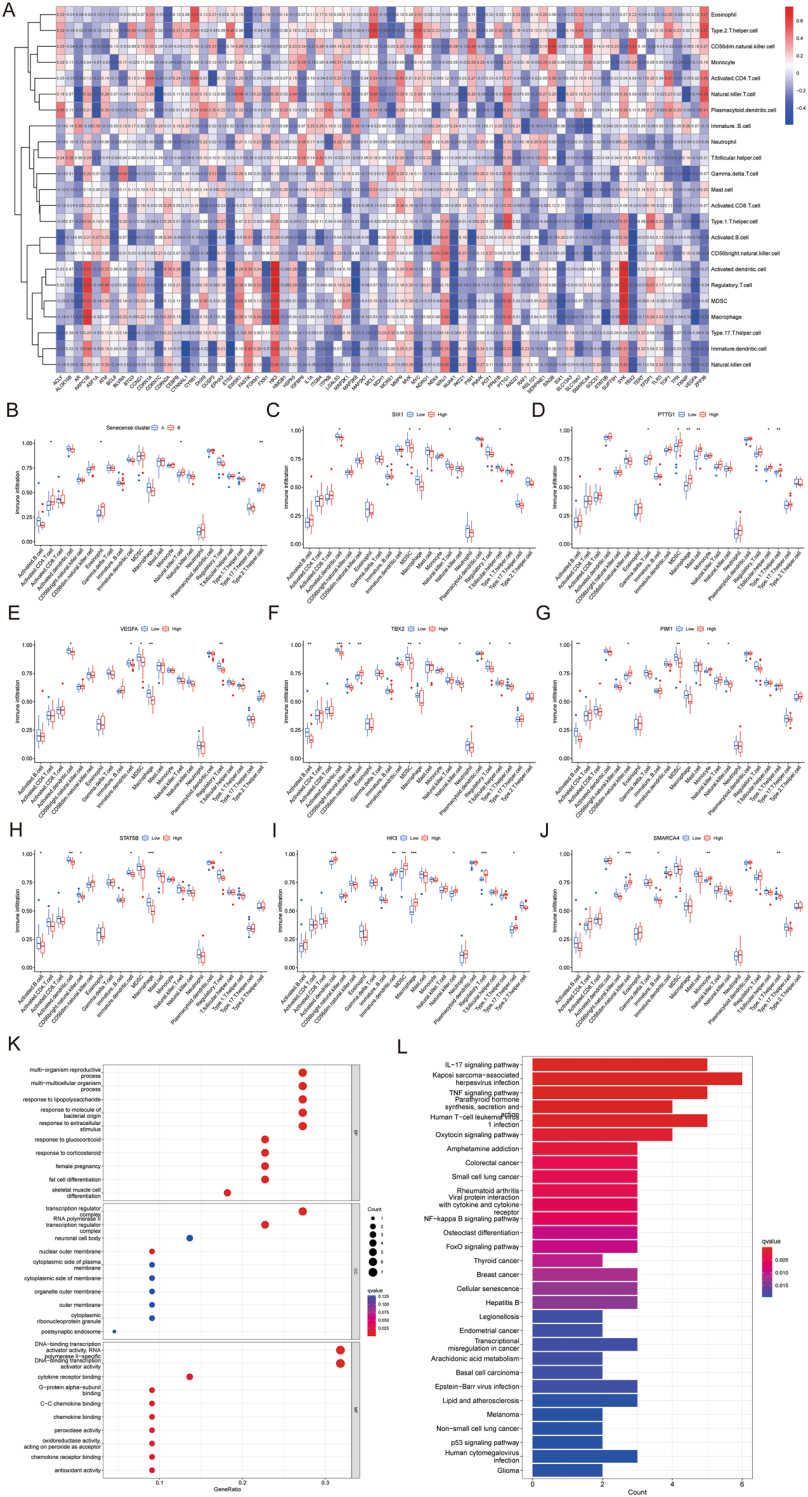
Immune cell infiltration and function enrichment analyses. (**A**) Heatmap showing the correlation between the expression of the 73 senescence regulators and immune cells infiltration using the ssGSEA method. (**B**) Boxplot showing the infiltrating immune cells in the two senescence clusters. (**C**–**J**) Correlation between key genes (SIX1, PTTG1, VEGFA, TBX2, PIM1, STAT5B, HK3 and SMARCA4) and immune cell infiltration. (K) The bubble diagram showing the top 10 terms of GO categories of BP, MF and CC. (**L**) Barplot diagram showing the KEGG enrichment analysis. (All figures *represents *p* < 0.05, **represents *p* < 0.01, ***represents *p* < 0.001) ssGSEA, single-sample gene-set enrichment analysis; GO, gene ontology; KEGG, Kyoto Encyclopedia of Gene and Genome; BP, biological process; MF, molecular function; CC, cellular component.

A more significant infiltration was found in activated CD4+ T cell (*P* < 0.05), eosinophil (*P* < 0.05), natural killer (NK) T cell (*P* < 0.05) and type 2 T helper cell (*P* < 0.01) (Fig. 4A,B). PTTG1, HK3 and SMARCA4 were positively related to immune cell infiltration, while SIX1, VEGFA, TBX2, PIM1 and STAT5B were negatively connected (Fig. 4C–J).

### Function enrichment analyses of senescence types

GO annotation and KEGG pathway analyses were conducted on DEGs between the two senescence clusters. The biological process (BP) of GO term result emphasized multi-organism reproductive process, multi-multicellular organism process, response to lipopolysaccharide, response to molecule of bacterial origin, response to extracellular stimulus, response to glucocorticoid, response to corticosteroid, female pregnancy, fat cell differentiation, skeletal muscle cell differentiation; for the molecular function (MF) of GO term, DEGs were significantly enriched in transcription regulator complex, RNA polymerase II transcription regulator complex, neuronal cell body, nuclear outer membrane, cytoplasmic side of plasma membrane, cytoplasmic side of membrane, organelle outer membrane, outer membrane, cytoplasmic ribonucleoprotein granule and postsynaptic endosome; the cellular components (CC) terms highlighted that DEGs were mainly concentrated on DNA-binding transcription activator activity, RNA polymeraseII-specific DNA-binding transcription activator activity, cytokine receptor binding, G-protein alpha-subunit binding, C–C chemokine binding, chemokine binding, peroxidase activity, oxidoreductase activity acting on peroxide as acceptor, chemokine receptor binding and antioxidant activity (Fig. 4K). The KEGG result manifested that DEGs were found to be mostly related to IL-17 signaling pathway, TNF signaling pathway oxytocin signaling pathway, rheumatoid arthritis, NF-kappa B signaling pathway, FOXO signaling pathway and so on (Fig. 4L).

### Generation of two senescence gene clusters and construction of senescence score

**Figure 5:**
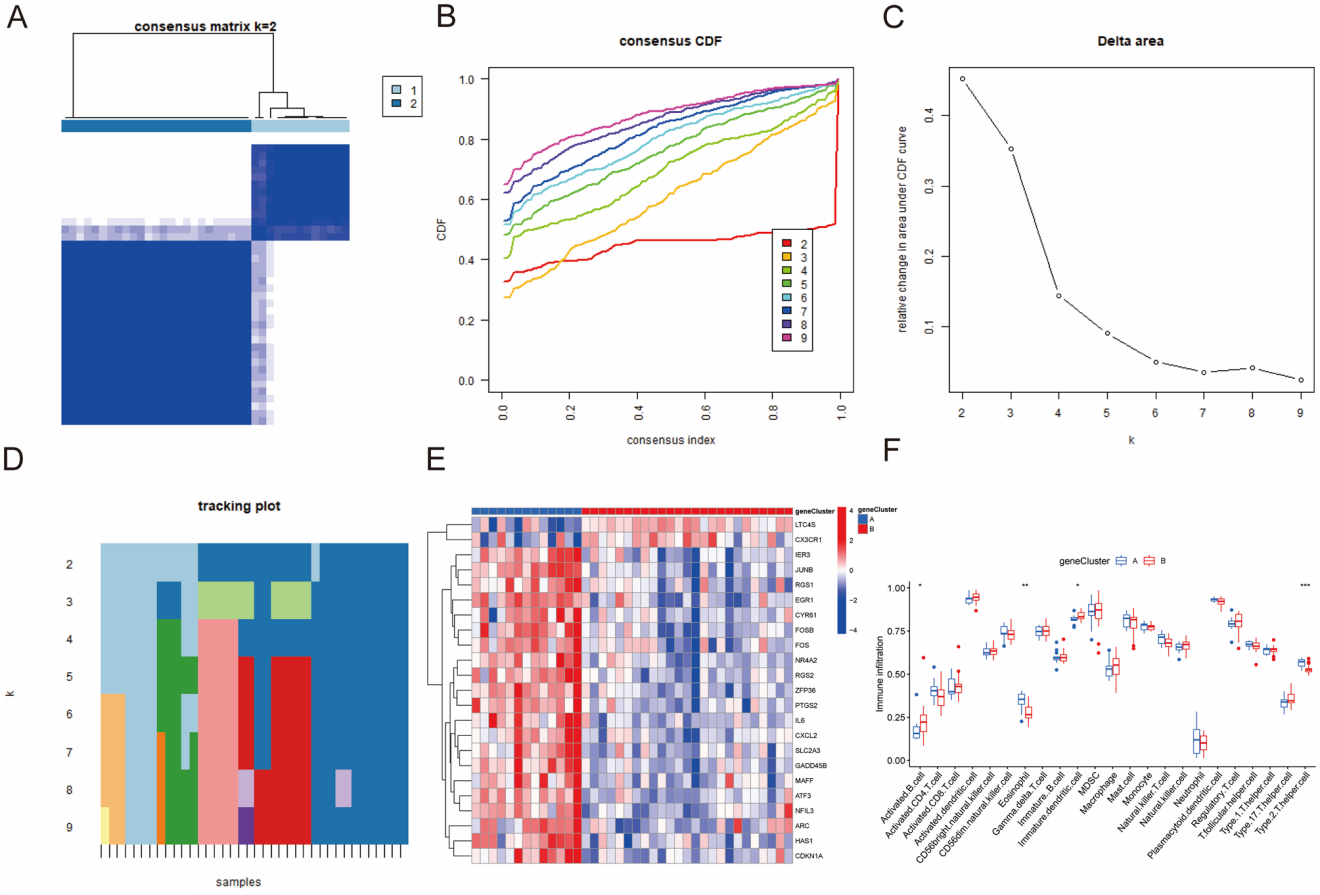
Clarification of two senescence gene clusters. (**A**–**D**) Clustering of OA synovial samples based on DEGs of the two senescence clusters. (**E**) Heatmap showing expression of DEGs in the two gene clusters. (**F**) Immune cell infiltration in the two gene clusters. (All figures *represents *p* < 0.05, **represents *p* < 0.01, ***represents *p* < 0.001) OA, osteoarthritis; DEGs, differentially expressed genes.

To further investigate senescence expression patterns, we generated gene clusters and constructed a scoring system. Consensus clustering results based on DEGs in the two senescence groups classified the patients into two gene clusters (gene cluster A and gene cluster B) (Fig. 5A–D). The heatmap showing that DEGs between gene cluster A and B is demonstrated in Fig. 5E.

**Figure 6:**
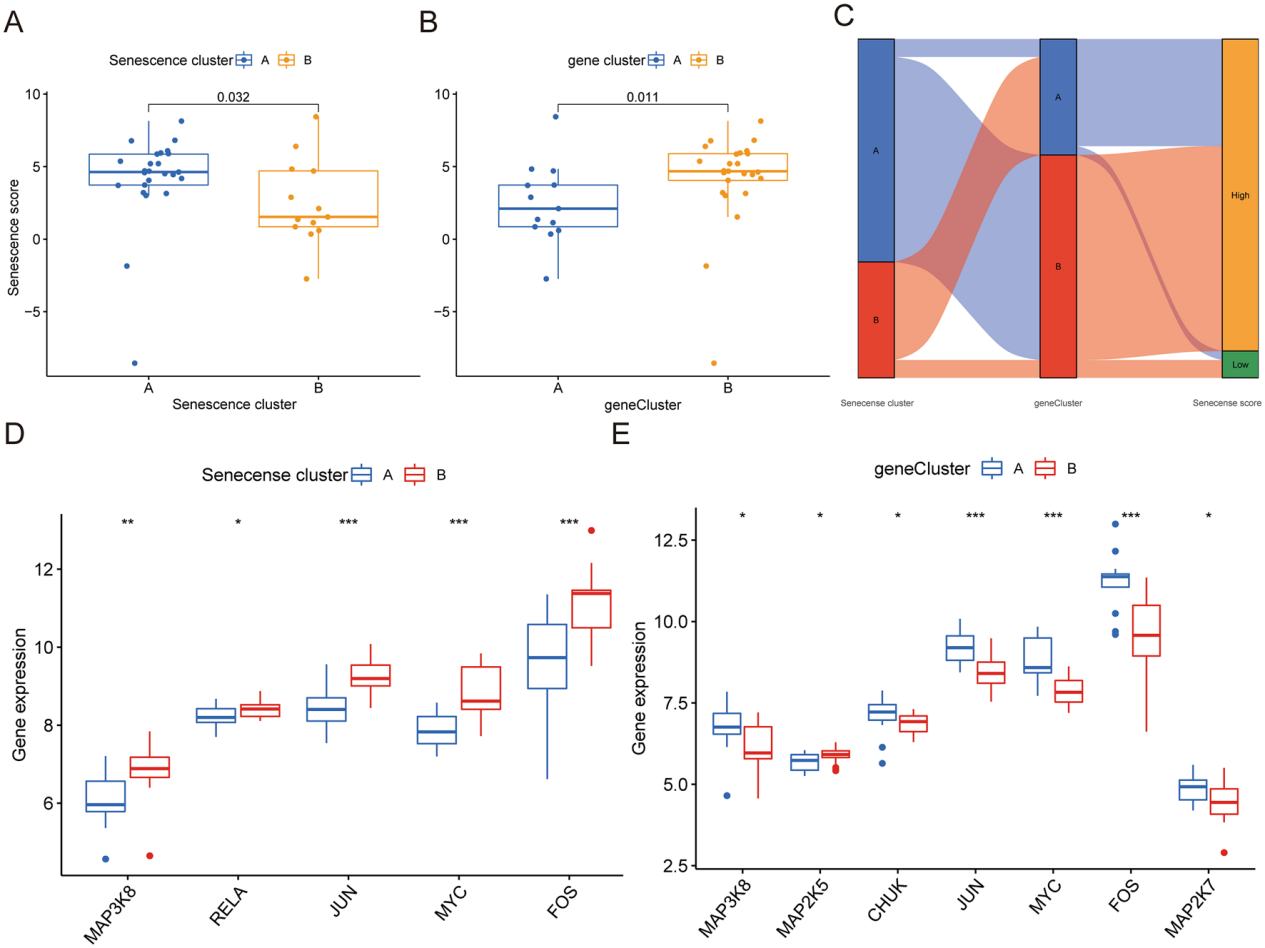
Construction of senescence signatures. (**A**) The senescence score in the two senescence clusters. (**B**) The senescence score in the two gene clusters. (**C**) Alluvial diagram showing the changes of senescence clusters, gene cluster and senescence score. (**D**) Bloxplot showing the gene expression of MAPK pathway in the two senescence clusters. (**E**) Bloxplot showing the gene expression of MAPK pathway in the two senescence gene clusters. (All figures *represents *p* < 0.05, **represents *p* < 0.01, ***represents *p* < 0.001) MAPK, mitogen-activated protein kinases.

We then constructed the senescence score using PCA based on DEGs between OA and normal. Next, we found that senescence score correlated with senescence (*P* = 0.032) and gene clusters (*P* = 0.011) (Fig. 6A,B); senescence cluster A and gene cluster B had higher senescence scores. Changes in individual patients among the GSE1919, GSE41038, GSE55235, GSE82107 and GSE55457 dataset were visualized in an alluvial diagram (Fig. 6C).

### Immune infiltration of senescence gene signatures

Figure 5F showed that activated B cell (P < 0.05), eosinophil (*P* < 0.01) and type 2 T helper cell (*P* < 0.001) infiltrated in gene cluster A more densely. Then we analyzed gene expression of MAPK pathway which is most related to inflammation in OA. Figure 6D displayed that MAP3K8 (*P* < 0.01), PELA (*P* < 0.05), JUN (*P* < 0.001), MYC (*P* < 0.001) and FOS (*P* < 0.001) in senescence cluster B. Figure 6E displayed that MAP3K8 (*P* < 0.05), MAP2K7 (*P* < 0.05) CHUK (*P* < 0.05), JUN (*P* < 0.001), MYC (*P* < 0.001) and FOS (*P* < 0.001) in gene cluster A.

## Discussions

OA is the most common joint disorder which involves biomechanics, inflammation, and complex biological responses of the immune system^35^. Although risk factors such as joint injury, obesity and genetics have all been linked to OA, the most prevalent risk factor is age^36^. There were several studies that suggested that underlying age-related changes could increase systemic and local inflammation and contribute to the development of osteoarthritis^10^. Local changes within the joint have been associated with increased production of inflammatory mediators and the secretion of the SASP^10^. However, researches about distinct senescence phenotypes of OA and their correlation with immune response is still blank in this field. In this study, we systematically investigated the senescence patterns in the OA immune microenvironment of synovial tissues.

Firstly, we observed a significant difference in the expression of 73 senescence regulator factors between synovial samples of OA patients and normal controls. Consistent results were obtained via RT-qPCR, which validated our findings. These results indicated that senescence regulators may be used as predictors of OA and involved in OA development. Based on the review of relevant literature, we found that nomograms are commonly used to predict OA occurrence and progression^37,38^. Similarly, we established a senescence nomogram for predicting the risk of OA from the perspective of cell senescence. This reaffirmed the important role of senescence regulatory factors in OA. Different scores were assigned to factors such as BCL6 and VEGFA. The total score was obtained by adding the scores of each factor. The total score was less than 35, the probability of OA was less than 0.1, and probability of OA was greater than 0.9 if the total score was greater than 75.

Moreover, we investigated the association between senescence regulatory factors and the immune properties of OA, including the gene set for immune cell infiltration and immune response. Unsupervised clustering of OA samples using senescence regulator expression profiles led to two subtypes with distinctive senescence patterns. Furthermore, the DEGs from 2 senescence clusters were further used to classify gene clusters by consensus clustering and 73 senescence DEGs was used to generate senescence scores for every patient by PCA. Most patients from senescence cluster B were further classified into gene cluster A and low m6A score group; whereas patients from m6A cluster A were classified into gene cluster B and high m6A score group. Our results also indicated that immune cells infiltrated more in senescence cluster B and gene cluster A, and gene expression of MAPK pathway which is the mostly related inflammation mechanism in OA were higher in senescence cluster B and gene cluster A^39^. Considering that each subtype has its immune characteristics, we believe that classification based on immunophenotypes of different senescence modulators is feasible and will help in comprehensively understanding the mechanisms of immune regulation.

Notably, *BCL6* and *VEGFA* were upregulated in OA tissues. Follicular helper T cells which is located in the follicles of lymphoid tissue, induce B cells to produce immunoglobulins and express various distinguishing genes such as B-cell lymphoma 6 (*BCL-6*)^40^. A study showed that OA patients showed higher percentages of follicular helper T cell and the expression of the cells was positively correlated with the disease activity of OA^41^. Additionally, numerous studies have also suggested that vascular endothelium growth factor A (*VEGFA*) plays an important role in cartilage development and OA progression^42^. Hamilton et al.’s research demonstrated that the expression of *VEGFA* in the synovium, articular cartilage and synovial fluid has been found to be significantly correlated with the grade of OA severity and the degree of pain^43^. These evidences confirmed that *BCL6* and *VEGFA* may be used as a predictive biomarker of OA.

Moreover, our study demonstrated that there was a significant difference of CD4+ T cell, type 2 T helper cell, NK T cell infiltration and the expression of IL17 pathways genes in the two distinct senescence clusters. T cells are the main components of synovial infiltration in OA patients, and both CD4+ T cells and CD8+ T cells have been found in synovial aggregates^40^. When stimulated by IL-4, naïve CD4+ T cells differentiate into type 2 T helper cells^40^. NK cells are a principal tissue-infiltrating lymphocyte subset in OA inflammation, and exhibit a quiescent phenotype consistent with post-activation exhaustion^44^. Type 17 T helper cells (Th17) secrete IL17 and provide protection against bacterial infections and are associated with the development of autoimmune diseases through recruitment of granulocyte cells, particularly neutrophils^40^. Existed researches demonstrated that Th17 cells accumulated in the synovial fluid and synovial tissue of OA patients; however, the exact role of Th17 cell response in the biology of OA needs further investigation^40^. Therefore, we assumed that different senescence modes can affect the immune microenvironment of OA, thus influencing the occurrence and development of OA.

However, there are still some limitations in our study that need to be pointed out. Firstly, control samples were all from patients with relatively normal synovial tissues, such as those undergoing surgery for meniscus injury, rather than real healthy controls. Secondly, due to the epidemiologic feature of OA patients, it was inevitable that there were age differences between OA samples and controls. Furthermore, no multiplicity was applied in our study to adjust the P value of each of the DEGs.

In conclusion, our study revealed a potential mechanism of senescence regulation in the immune microenvironment of OA synovial tissues. We revealed two distinct senescence patterns, identified a strong correlation between different senescence patterns and immune cell infiltration, and constructed a novel scoring system to quantify senescence pattern in individual patients. Meanwhile, the developed senescence OA nomogram can help assess the risk of OA, thus providing a reference for the clinical diagnosis of OA. Our findings provided novel ideas for identifying different OA inflammatory phenotypes, promoting personalized immunotherapy, and opening up new horizons for future research on the pathogenesis of OA.

## Supplementary Information

Supplementary Information.

## Data Availability

The datasets presented in this study were obtained from the GEO (https://www.ncbi.nlm.nih.gov/geo/) (GSE1919, https://www.ncbi.nlm.nih.gov/geo/query/acc.cgi/; GSE41038, https://www.ncbi.nlm.nih.gov/geo/query/acc.cgi/; GSE55235, https://www.ncbi.nlm.nih.gov/geo/query/acc.cgi/; GSE82107, https://www.ncbi.nlm.nih.gov/geo/query/acc.cgi/; GSE55457, https://www.ncbi.nlm.nih.gov/geo/query/acc.cgi/).
