# Supplementary material for: Identification and validation of BCL6 and VEGFA as biomarkers and ageing patterns correlating with immune infiltrates in OA progression
Source: Sci Rep. 2023 Feb 13;13:2558. doi: 10.1038/s41598-023-28000-9 (PMC9925801; doi:10.1038/s41598-023-28000-9)
Supplement: Supplementary file 1 — Supplementary Information. [file 41598_2023_28000_MOESM1_ESM.pdf]

Table S1 Primers employed in this study

Gene symbol	Forward primer (5'-3')	Reverse primer (5'-3')
BCL6	GCAGACCCACAGTGACAAACC	TCCGCAGGTTTCGCATTTG
VEGFA	GGAGGGCAGAATCATCACGA	GCTCATCTCTCCTATGTGCTGG
